# Obstacle Avoidance Strategy and Path Planning of Medical Automated Guided Vehicles Based on the Bionic Characteristics of Antelope Migration

**DOI:** 10.3390/biomimetics10030142

**Published:** 2025-02-26

**Authors:** Jing Hu, Junchao Niu, Bangcheng Zhang, Xiang Gao, Xinming Zhang, Sa Huang

**Affiliations:** 1 School of Mechatronical Engineering, Changchun University of Science and Technology, Changchun 130022, China; 2 Changchun Institute of Technology, Changchun 130103, China; 3 School of Mechatronic Engineering and Automation, Foshan University, Foshan 528225, China; 4 Precision Machining and Special Machining Innovation Team, Guangdong Education Department, Foshan 528225, China; 5 Bethune Second Clinical School of Medicine, Jilin University, Changchun 130015, China

**Keywords:** medical AGV, bionic strategy, artificial potential field algorithm, dynamic window algorithm, path planning

## Abstract

Automated Guided Vehicles (AGVs) face dynamic and static obstacles in the process of transporting patients in medical environments, and they need to avoid these obstacles in real time. This paper proposes a bionic obstacle avoidance strategy based on the adaptive behavior of antelopes, aiming to address this problem. Firstly, the traditional artificial potential field and dynamic window algorithm are improved by using the bionic characteristics of antelope migration. Secondly, the success rate and prediction range of AGV navigation are improved by adding new potential field force points and increasing the window size. Simulation experiments were carried out on a numerical simulation platform, and the verification results showed that the bionic obstacle avoidance strategy proposed in this paper can avoid dynamic and static obstacles at the same time. In the example, the success rate of path planning is increased by 34%, the running time is reduced by 33%, and the average path length is reduced by 1%. The proposed method can help realize the integration of “dynamic and static” avoidance in the process of transporting patients and effectively save time by using AGVs to transport patients. It provides a theoretical basis for realizing obstacle avoidance and rapidly loading AGVs in medical environments.

## 1. Introduction

An AGV (Automated Guided Vehicle) is an automatic guided vehicle capable of autonomous navigation without human intervention [1], and they have been widely used in logistics [2], medicine [3], shipping [4], industry [5], and other scenarios. With the rapid development of AGV technology [6], AGVs have gained increasing attention in the medical field as automated transport equipment, and they are gradually developing in the direction of intelligence [7]. Therefore, it is a key research direction for AGVs to safely avoid dynamic and static obstacles, such as pedestrians and medical devices in the medical environment [8], and to reasonably plan a path from the starting point to the target point [9].

At present, scholars from different countries are conducting a range of research on and are making a series of improvements to the path planning algorithm and the limitations of the algorithm itself [10]. For robots applied in the marine environment, Hou et al. [11], Yang et al. [12], and Singh et al. [13] respectively proposed methods of adding the distance penalty factor, setting adaptive guidance angles, and improving safety distance to solve the problem of the influence of ocean current and wind direction on the safety distance of marine robots. For robots applied in the orchard environment, Kang et al. [14] and Zhang et al. [15] respectively proposed a bidirectional path planning algorithm based on the random tree algorithm and an improved A* algorithm. By simulating the kinematics model of a lawn mower, the number of turns in path planning was reduced, and the problems of task assignment and battery life were effectively solved. Differing from the former optimization idea that considers battery life, Zhang et al. [16] believe that the complex orchard environment is more likely to affect the efficiency of path planning. They reduced the complexity of the potential field by modifying the range of the repulsive field. They also made the distance of path planning shorter, aiming to solve problems such as vehicle degree, safety verification capability, and path planning in the process of automatic driving. Qin et al. [17], Zhang et al. [18], and Zhao et al. [19] respectively proposed three improvement strategies: improving the artificial potential field algorithm, combining the A* algorithm and random tree algorithm, and optimizing traditional particle swarm optimization. Through the hierarchical solution of the original algorithm and the simplified map, the solution time was shortened, and the obstacle avoidance ability and real-time performance of the unmanned vehicle were enhanced. To solve the problem of the poor dynamic performance of the traditional artificial potential field algorithm, Jin et al. [20] introduced a rotation factor to reduce the calculation burden and adopted a gradient descent method to guide vehicle motion. In a different way than the former, Lee et al. [21] proposed a virtual mountain algorithm, which reduces the error in algorithm operation and also improves the efficiency of calculation. To solve the local optimization problem of the traditional particle swarm optimization algorithm, Tao et al. [22] divided the particles of the original algorithm into two populations to increase the random disturbance and diversity of particles and optimized the path smoothness based on the B-spline curve method. Similarly, in dealing with the improvement of the particle swarm optimization algorithm, Huang et al. [23] gave different strategies. Their team came up with a balanced power model that improved the stability of the particle swarm algorithm. In order to avoid dynamic obstacles, Liao et al. [24] put forward a path planning method that integrates the adaptive A* algorithm and improves the dynamic window method. Hou et al. [25] combined the enhanced sparrow search algorithm and dynamic window method to solve the problems of over traversal of nodes and local optimality, respectively. In terms of algorithm optimization and control, Hami et al. [26] used the variational method to optimize the control of four-wheel steering vehicles, and Zheng et al. [27] designed a double-loop trajectory control strategy, both of which realized a good obstacle avoidance strategy, laying a foundation for robots to work in complex scenes.

From the above studies, it can be observed that most current research on obstacle avoidance strategies focuses on either improving the algorithm itself or studying dynamic and static obstacles, while there are a few studies on the combination of “dynamic and static” algorithms. The accurate and effective implementation of the “dynamic-static” integrated obstacle avoidance strategy in medical patient transport AGVs can enhance transport efficiency and save valuable time for patient treatment. Targeting the combination of unpredictable pedestrians, medical equipment, and other static and dynamic obstacles encountered by medical transport AGVs, this paper proposes a bionic “dynamic-static” integrated obstacle avoidance strategy inspired by the adaptive behavior of antelopes during migration, improving the traditional artificial potential field and dynamic window algorithms [28]. The limitations of the current obstacle avoidance algorithm used in AGV path planning have been addressed, and obstacle simulation experiments with varying complexities have been conducted on a numerical simulation platform to demonstrate the feasibility of the proposed algorithm, thereby providing a theoretical foundation for the application of intelligent obstacle avoidance in patient-loaded AGVs.

## 2. Selection of a Bionic Strategy Prototype and Algorithm

Unlike outdoor environments such as oceans and mountains, mobile AGVs face distinct obstacle avoidance challenges in medical environments, such as medical personnel with unpredictable routes, a variety of large medical equipment, and densely populated areas like outpatient departments and registration offices, which are prone to congestion. At the same time, many animals in nature exhibit bionic behaviors that can be reflected in the path planning strategies of AGVs. Therefore, the selection of a bionic model and algorithm should not only ensure the similarity between the bionic prototype and path planning behavior but also ensure the flexibility and intelligence of the algorithm. Only by choosing a path planning algorithm suitable for the medical environment can reliable obstacle avoidance navigation be realized in the real-world environment that combines both static and dynamic elements.

## 3. Prototype of the Bionic Strategy

Antelopes are medium-sized, even-toed ungulates known for their agility in running and excellent eyesight. They are found throughout Africa and Asia and can adapt to a variety of ecosystems, including grasslands, deserts, mountains, and forests.

**Figure 1:**
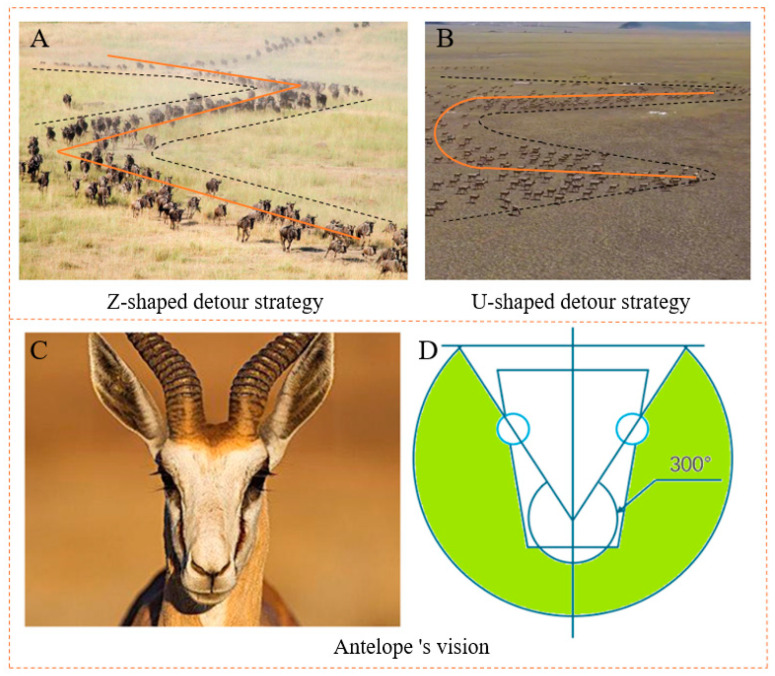
Bionic behavior of antelopes: (**A**) Z-shaped detour strategy; (**B**) U-shaped detour strategy; (**C**,**D**) antelope’s vision.

Migration, as one of the key survival strategies of antelopes, exhibits biomimetic behaviors that may inspire and guide the navigation of medical patients using AGVs. These behaviors are illustrated in Figure 1.

(1) Antelopes exhibit unique detour strategies during migration, enabling them to consider the types and distribution of obstacles, thereby guiding their decisions on future migration routes. The antelope circumvents fixed obstacles to find a new target point. These new target points are not only fixed points on the shortest path but also flexible and safe target points selected through circumnavigation thinking. This detour idea provides a kind of optimization and trade-off approach in path planning; we can evaluate the distance between different target points and assess whether the AGV can pass safely, thus enabling us to choose the most suitable path.
(2) The antelope has a conical field of vision, with its eyes capable of observing nearly 300° ahead. This unique visual feature enables the antelope to effectively detect obstacles both ahead and to the side during migration. By leveraging this conical vision, the antelope can fully assess the width and openness of a feasible path, avoiding potential obstacles or safety risks by selecting a wider route.

Based on the bionic behavior of antelopes during migration, this paper proposes an integrated “dynamic and static” obstacle avoidance method inspired by their migration strategy. The algorithm is tailored and improved for medical environments, focusing on enhancing operational time and path planning robustness, so that the new strategy can better adapt to complex medical environments and achieve more effective path planning.

## 4. Selection of the Path Planning Algorithm

**Figure 2:**
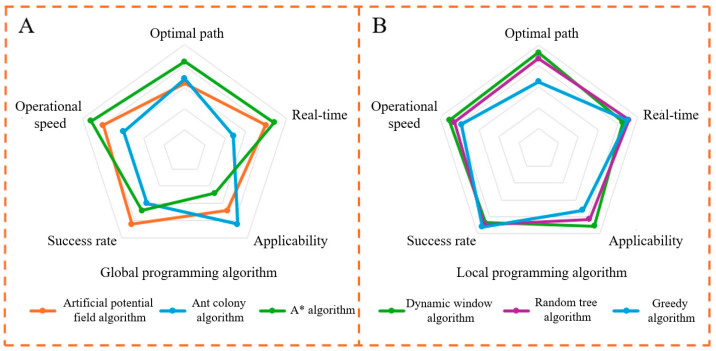
Advantages and disadvantages of the algorithm: (**A**) global programming algorithm and (**B**) local programming algorithm.

The existing path planning methods can be divided into global path planning [29] and local path planning [30]. Commonly used algorithms include the ant colony algorithm [31], A* algorithm [32], random number algorithm [33], greedy algorithm, etc. In this paper, five evaluation indexes that are more important in the medical environment are used as the scoring criteria for the algorithm radar map. The performance of different algorithms in each index is shown in Figure 2.

A and B in Figure 2 represent the advantage curves of different algorithms for global and local path planning, respectively. The artificial potential field algorithm was applied to robotic arms and robots [34]. After hierarchical optimization, the algorithm demonstrated strong adaptability, while the dynamic window algorithm provided excellent real-time performance, effectively handling sudden dynamic obstacles. Since the medical AGV operates in environments with both dynamic and static obstacles, such as doctors and pedestrians whose motion states change during operation, it is essential to minimize load and transport time while avoiding obstacles in complex scenarios. Considering the algorithm’s speed requirements, the detour strategy and conical visual range perception ability observed in antelope migration are incorporated into the obstacle avoidance strategy. The artificial potential field algorithm and dynamic window algorithm are applied to global and local path planning, respectively.

## 5. Algorithm Problems and Improvements

### 5.1. Artificial Potential Field Algorithm

Based on the concept of the potential field in physics [35], the artificial potential field algorithm models the robot and the environment as a potential field system, determining the moving direction of the robot by calculating and optimizing the potential field.

The potential field function is defined as the sum of the gravitational field and the repulsive field, and the function expression is shown as Equation (1):

(1) $$U_{t}\left(X\right)=U_{a}\left(X\right)+U_{r}\left(X\right)$$

Among them, $U_{t}$ represents the sum of potential field forces, $U_{a}$ represents the gravitational field, $U_{r}$ represents the repulsive force field, and X(x,y) represents the AGV position vector.

The calculation formula of the gravitational field is shown in Equation (2):

(2) $$U_{a}\left(X\right)=K_{a}{\left(X-X_{g}\right)}^{2}/2$$

Among them, $K_{a}$ represents the gravitational gain constant and $X_{g}(x_{0},y_{0})$ represents the target point position vector.

The calculation formula of the repulsive force field is shown in Equation (3):

(3) $$U_{r}\left(X\right)=\left\{\begin{array}{c} K_{r}{\left(\frac{1}{p_{o}}-\frac{1}{p}\right)}^{2}{\left(X-X_{g}\right)}^{n}/2\;\;\;\;\;p_{o}<p\\ \;\;\;\;\;0\;\;\;\;\;p_{o}\geq p \end{array}\right.$$

Among them, $K_{r}$ represents the repulsive force gain constant, p represents the maximum influence distance of a single obstacle and its evaluation criterion is typically based on the physical characteristics of the obstacle, $p_{o}$ represents the shortest distance between AGV and the obstacle, and n represents an exponent factor used to adjust the intensity of the obstacle avoidance force field, with a range of 0 ≤ n ≤ 1.

The expression of the formula is shown in Equation (4):

(4) $$p_{o}\;=\;X-X_{o}$$

Among them, $X_{o}(x_{1},y_{1})$ indicates the obstacle position vector.

The force AGV receives in the potential field is the sum of the gravitational and repulsive forces, and the specific expression is shown in Equation (5):

(5) $$F_{t}\left(X\right){\;=\;F}_{a}\left(X\right)+F_{r}\left(X\right)$$

Among them, $F_{t}$ represents the resultant force on the AGV, $F_{a}$ represents the gravitational force, and $F_{r}$ represents the repulsive force.

The calculation formula for the gravitational force is shown in Equation (6):

(6) $$F_{a}\left(X\right)=-K_{a}\left(X-X_{g}\right)$$

The calculation formula for repulsion is shown in Equation (7):

(7) $$F_{r}=\left\{\begin{array}{c} K_{r}\left(\frac{1}{p_{o}}-\frac{1}{p}\right)\cdot \left(\frac{1}{p_{o}^{2}}\right)\cdot \left(\frac{\partial p_{o}\left(X\right)}{\partial X}\right)\;\;\;\;p_{o}<p\\ \;\;\;\;\;\;\;\;\;\;\;\;0\;\;\;\;\;\;\;\;\;\;\;\;p_{o}\geq p \end{array}\right.$$

However, under the influence of certain complex potential field forces, when a mobile robot moves to a point on the map, its repulsive force becomes equal to the gravitational force, causing the resultant force to become zero and the robot to stop moving. This phenomenon is a common local minimum problem in traditional artificial potential field algorithms.

**Figure 3:**
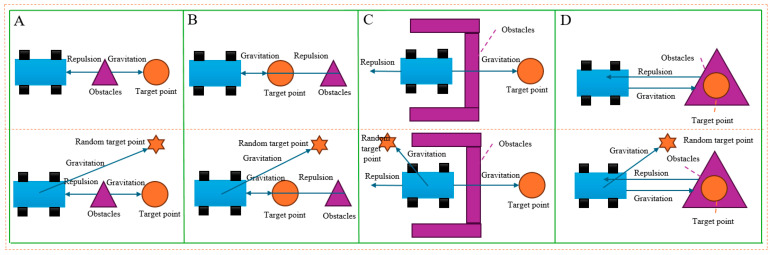
Improvement of the artificial potential field algorithm: (**A**,**B**) “Line” type obstruction; (**C**) U-shaped obstacle; (**D**) “Encirclement” type obstacle.

Figure 3 shows that during the robot’s movement, the obstacle and the target point lie on the same straight line. At this point, the gravitational and repulsive forces are equal in magnitude and opposite in direction, resulting in a zero resultant force acting on the robot, causing it to stop moving. Figure 3C shows that when the robot encounters a U-shaped obstacle, due to the complex force situation, the robot becomes trapped in the area and is unable to reach the target point, Figure 3D shows that when the target point is within the range of the obstacle, the robot is pushed away from the target point due to the excessive repulsion from the obstacle.

In the early 1990s, in the area of traffic management, the vector potential field method was used to address the local minimum problem in the artificial potential field algorithm [36]. The approach involved adding a perpendicular component at the local minimum points to disperse the potential field forces, thereby preventing the robot from getting trapped in a local optimum. Similarly, during migration, antelopes exhibit a detour strategy by observing their environment and adjusting their direction flexibly to find more adaptable target points when faced with complex obstacle distributions. This strategy inspires us to recognize that the local optimal solution problem faced by AGV navigation in the artificial potential field algorithm also arises from the relative position between obstacles and the target point. Therefore, similar adjustment methods are necessary to ensure that the robot can effectively navigate around obstacles and continue its movement.

There are already many solutions to the local minimum problem in the artificial potential field algorithm. Yu et al. [37] improved the local minimum problem by proposing a hybrid heuristic function approach. Liu et al. [38] designed new attractive and repulsive potential functions and developed a new dynamic collision avoidance process to solve this problem. Most of the above methods improve the original algorithm by establishing new functions, which are relatively complex to implement and require the consideration of many parameter factors during function design.

This paper addresses the local minimum problem of mobile robots by adjusting the target point position, mimicking the bionic behavior of antelopes during migration. It adds a random target point to the left of the original target point and applies additional force through this random target point to disrupt the original force balance, helping the robot escape the local minimum and continue moving toward the target point. At the same time, the gravitational force is attenuated, and the maximum value of the gravitational gain parameter $K_{a}$ is restricted to prevent the attractive force from becoming too large, which could cause the robot to move rapidly and increase the risk of collision with obstacles. The force acting on the random target point is shown in Figure 3.

After adding random target points, the AGV will be subjected to the additional gravitational force of the new target points, and the new resultant force is shown in Equation (8):

(8) $$F_{t}\left(X\right)\;=\;F_{a}\left(X\right)+F_{r}\left(X\right)+F_{n}\left(X\right)$$

Among them, $F_{n}$ represents the gravitational attraction of the new target point.

After the random target points are generated, the simulated annealing process is used for navigation, and the specific steps are as follows: At the current local minimum point X(x,y), select a random point target point $X_{0}(x_{0},y_{0})$ and then calculate the potential fields $U_{t}(X)$ and $U_{t}(X_{0})$ at points X and $X_{0}$ respectively. If $U_{t}\left(X_{0}\right)\leq U_{t}(X)$ is met, $X_{0}$ is accepted as the next point. If $U_{t}\left(X_{0}\right)$ > $U_{t}(X)$ is satisfied, then this point is accepted as the next point with probability l, where, the probability l comes from the Metropolis–Hastings algorithm [39] criterion of the inner loop of the simulated annealing algorithm, and the specific expression is shown in Equation (9):

(9) $$l=\left\{\begin{array}{c} exp\left(-\frac{E\left(X_{n}\right)-E\left(X_{o}\right)}{T}\right)\;\;E\left(X_{n}\right)>E\left(X_{o}\right)\\ \;\;\;\;\;\;\;\;\;1\;\;\;\;\;\;E\left(X_{n}\right)\leq E\left(X_{o}\right) \end{array}\right.$$

Among them, $X_{n}$ represents the later time, $X_{o}$ represents the previous time, $E\left(X_{n}\right)$ represents the internal energy at $X_{n}$, $E\left(X_{o}\right)$ represents the internal energy at $X_{o}$, and T represents the temperature at this moment [40].

The temperature T inside the algorithm decreases as the operation time increases, and the specific expression is shown in Equation (10):

(10) $$T\left(t\right)=\alpha T\left(k-1\right)$$

Among them, α is a constant less than 1, usually in the range of (0.85, 1), and k is the number of iterations.

### 5.2. Dynamic Window Algorithm

**Figure 4:**
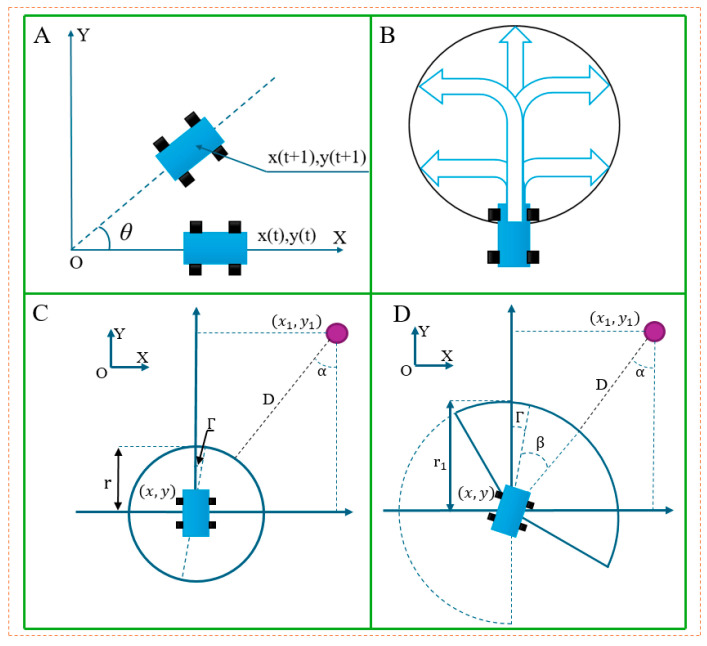
Improvement of the dynamic window algorithm: (**A**,**B**) the steering diagram of the car and (**C**,**D**) the improved dynamic window algorithm diagram.

The basic principle of the dynamic window algorithm is to treat the environment around the robot as a dynamic window, which constantly updates with the robot’s movement. The algorithm dynamically adjusts the robot’s path by detecting obstacles and environmental changes within the window, ensuring that it can safely and efficiently traverse complex environments. Figure 4 shows the movement model of the mobile robot at each time period t in the coordinate system XOY, which takes into account the velocity space [v(t), ω(t)] and state space [x(t),y(t)], including linear velocity $v\left(t\right)$, steering angle $\theta \left(t\right)$, angular velocity ω(t), and attitude angle Γ(t). At time t, the expression of the robot’s kinematic model is:

(11) $$\left\{\begin{array}{c} x\left(t+1\right)\;=\;x\left(t\right)+v\left(t+1\right)cos\theta \left(t\right)\cdot \Delta t\\ y\left(t+1\right)\;=\;y\left(t\right)+v\left(t+1\right)sin\theta \left(t\right)\cdot \Delta t\;\\ \Gamma \left(t+1\right)\;=\;\theta \left(t\right)+\omega \left(t\right)\cdot \Delta t \end{array}\right.$$

The traditional dynamic window algorithm is applied to the obstacle position with the shortest current position of the AGV. When the angular velocity and linear velocity remain constant, the window size is usually fixed. The robot’s motion trajectory is a circle, as shown in Figure 4B, and its radius expression is:

(12) $$r\;=\;\left|v\right|/\left|\omega \right|\;\;\left(\omega \neq 0\right)$$

The traditional obstacle distance evaluation function is:

(13) $$D\left(v,\omega \right)\;=\;\sqrt{{\left(x-x_{1}\right)}^{2}+{\left(y-y_{1}\right)}^{2}}-r$$

Among them, $D\left(v,\omega \right)$ represents the distance between the AGV and obstacle, and $X_{o}(x_{1},y_{1})$ represents the obstacle position vector.

Due to the fixed window size, for some unpredictable dynamic obstacles, when the medical AGV moves too fast, simply using r in the above equation as the safety distance may lead to accidents and collisions because of the short safety distance. Secondly, algorithms are sensitive to local minimum points in complex environments, which may lead to the robot becoming stuck in local optimal solutions and prevent it from finding the global optimal path.

To enhance the safety of medical AGVs and more effectively avoid dynamic obstacles, this paper proposes an improved obstacle distance evaluation function based on the conical field of view observed in antelopes. As shown in Figure 4, the sector radius $r_{1}$ is adopted as the safety distance evaluation index, and the length of sector radius $r_{1}$ is defined according to the velocity space of obstacles. The length expression for $r_{1}$ is shown in Equation (14):

(14) $$r_{1}=v_{o}\cdot \Delta t+r$$

Among them, $v_{o}$ is the speed of the moving obstacle in the obstacle avoidance environment, r is the radius of the original evaluation function. It can be seen from the above formula that the length of sector radius $r_{1}$ changes with the dynamic obstacle velocity. Compared with the evaluation function r of the traditional algorithm, the faster the speed of the dynamic obstacle, the longer the sector radius length will be.

In Figure 4D, the offset Angle α of the obstacle position relative to the current AGV position can be expressed as:

(15) α=Γ+β

Among them, Γ is the angle of the robot’s velocity with respect to the *y*-axis and β is the difference of the declination angle from the position of the obstacle.

In the improved dynamic window algorithm, under the joint action of linear velocity v and angular velocity ω, the projected position of the AGV on the long side of the sector after Δt time can be defined as $X_{c}(x_{3},y_{3})$. The modified obstacle evaluation function is shown in Equation (16):

(16) $$D\left(v,\omega \right)=\sqrt{{\left(x-x_{3}\right)}^{2}+{\left(y-y_{3}\right)}^{2}}-r_{1}$$

At the same time, in order to constrain the distance between the dynamic window algorithm and the key nodes in the global path, the distance evaluation subfunction point (v,ω) and the trajectory evaluation function $O_{r}\left(v,\omega \right)$ are added to the nodes. The specific expression of the distance evaluation subfunction is shown in Equation (17):

(17) $$point\left(v,\omega \right)=\min\left[\sqrt{{\left(x-x_{no}\right)}^{2}+{\left(y-y_{no}\right)}^{2}}\right]$$

Among them, X(x,y) is the current position coordinate, and $X_{no}(x_{no},y_{no})$ is the key node coordinate. The weight value of point(v,ω) is calculated to determine whether the path is offset. The smaller the weight value is, the more it fits the global planning path. The value of the trajectory evaluation function $O_{r}\left(v,\omega \right)$ is adjusted appropriately according to the subfunctions $\;D\left(v,\omega \right)$ and point(v,ω).

**Figure 5:**
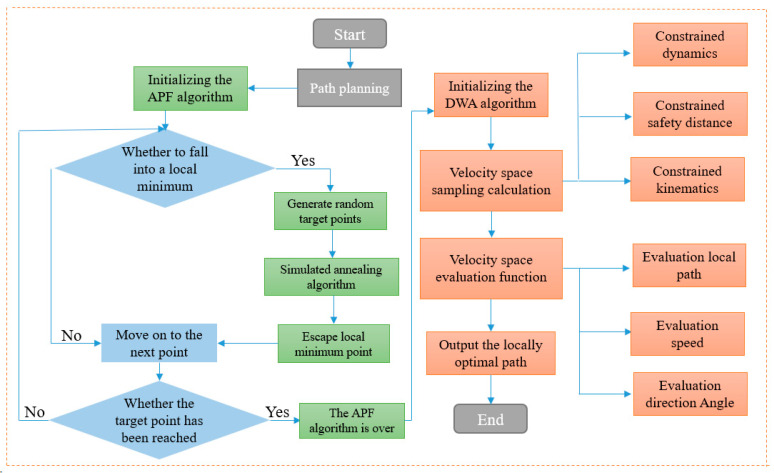
Improved algorithm flow based on the bionic strategy of antelope migration.

Figure 5 shows the flowchart of the fusion algorithm: the traditional artificial potential field and dynamic window algorithms are optimized based on the bionic behavior of antelope during migration. The circumferential strategy and conical sight distance, applied during navigation and migration, are incorporated into the algorithm. In global path planning, the improved artificial potential field algorithm is used to generate the obstacle avoidance path and extract key nodes. In local path planning, the AGV’s sensors collect surrounding environmental data, generate real-time map information, predict obstacle locations, and derive the local optimal path through speed and trajectory analysis.

## 6. Simulation Results and Data Processing

To verify the obstacle avoidance performance of the path planning algorithm, the improved algorithm was compared with the traditional one in a medical environment simulation on a numerical simulation platform.

## 7. Artificial Potential Field Algorithm Simulation Experiment

Firstly, global path planning is compared between the traditional artificial potential field algorithm and the improved artificial potential field algorithm. For the traditional artificial potential field algorithm, there are “obstacle points” that cause the algorithm to fall into a ‘local optimal solution when path planning is carried out on the map. Therefore, a 10 × 10 two-dimensional static grid map is set up, along with the starting point coordinates, the endpoint coordinates, the obstacle coordinates, the end point coordinates, and the obstacle coordinates that are likely to lead to a local optimal solution. The number of iterations is set to 100. The main internal parameters of the algorithm are shown in Table 1.

**Figure 6:**
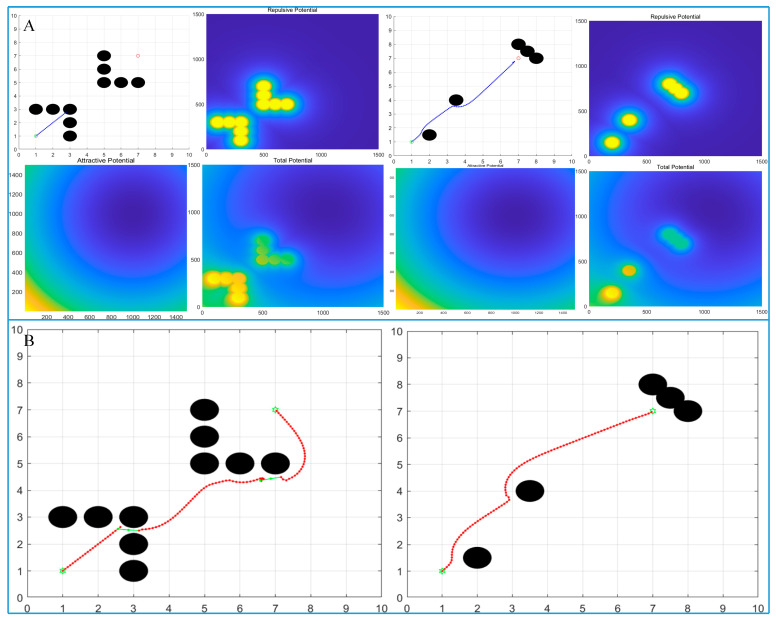
Comparison experiment of path planning: (**A**) traditional artificial potential field algorithm and (**B**) improved artificial potential field algorithm.

Figure 6 simulates the planned paths of the traditional algorithm and the improved algorithm under two different obstacle environments.

As shown in Figure 6A, the determination of obstacle coordinate points in two road conditions is quite detailed, and these obstacle coordinate points cause the traditional artificial potential field algorithm to fall into the local optimal solution, ultimately leading to the failure of path planning.

In Figure 6A, the obstacle in the first road condition is set as “U” shaped. Because the obstacle imposes a too repulsive force on the AGV via the potential field on the AGV, the traditional algorithm fails to reach the target point and halts the path planning at (3,3).

In the second road condition, obstacles are placed near the target point. It is clear that as the AGV approaches the target point, the surrounding obstacles exert excessive repulsive force on the AGV, resulting in the AGV stopping movement near the target point.

These two cases illustrate the limitations and drawbacks of the traditional algorithm. When the number of obstacles is large and the location of obstacles is relatively complex, it is difficult for the traditional method to achieve effective path planning.

Figure 6B shows the path planned by the improved artificial potential field algorithm. Unlike the traditional method, the improved algorithm can continue path planning by setting virtual target points owing to its strong robustness when it falls into a local optimal solution.

Based on the path planned by the algorithm, it can be seen that the improved artificial potential field algorithm successfully overcomes the local optimal solution problem and then plans a reasonable path for the AGV to safely reach the target point.

**Figure 7:**
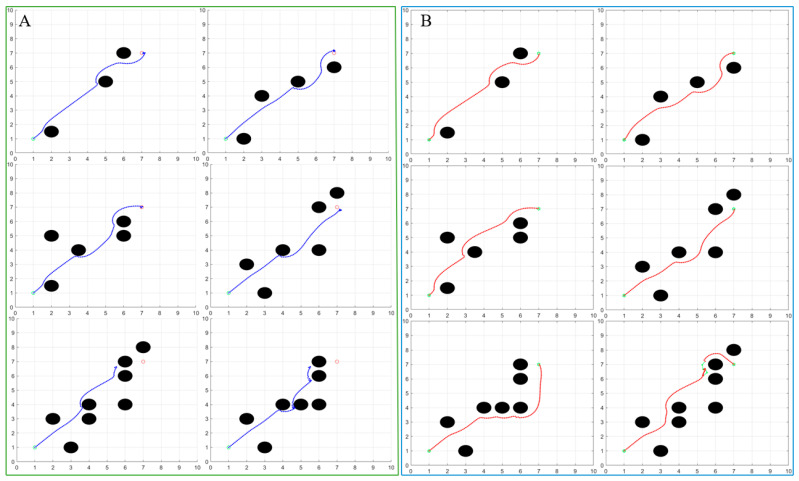
Comparison of the simulation results of the two algorithms: (**A**) traditional algorithm for path planning and (**B**) improved algorithm for path planning.

We have designed additional simulation experiments which clearly demonstrate the superiority of the improved algorithm. Figure 7 shows the comparison experiment between the traditional algorithm and the improved algorithm under six different road conditions.

For the traditional artificial potential field algorithm, the distance between the path and the obstacle is shorter when facing the road condition with simple obstacle distribution. This path increases the risk of an AGV colliding with an obstacle and creates several unsafe pitfalls. When confronted with a more complex distribution of obstacles, the traditional algorithm will be affected by multiple factors such as overly complicated potential field forces and imperfect algorithm design. These effects, as represented in the simulation model, result in the AGV not being able to reach the target point smoothly. Additionally, as the number of obstacles increases, the degree of trajectory deviation becomes larger and larger. However, the improved algorithm has better countermeasures in the face of the above problems. In the process of automatic navigation, the robot maintains a greater distance from obstacles, follows a smoother path, and has higher success in path planning.

The optimized potential field algorithm not only ensures the success rate of path planning but also reduces the average running time of the algorithm. Specific data are shown in Table 2.

## 8. Dynamic Window Algorithm Simulation Experiment

A 10 × 10 two-dimensional static grid map was set up on the numerical simulation platform, and the starting point (0,0) and end point (7,7) were set. The main parameters of the dynamic window algorithm are shown in Table 3.

The map modeling is the same as the scene in Figure 7. A random static obstacle and four dynamic obstacles are set up, and the dynamic obstacles move uniformly along the positive direction of the X axis and the positive direction of the Y axis.

**Figure 8:**
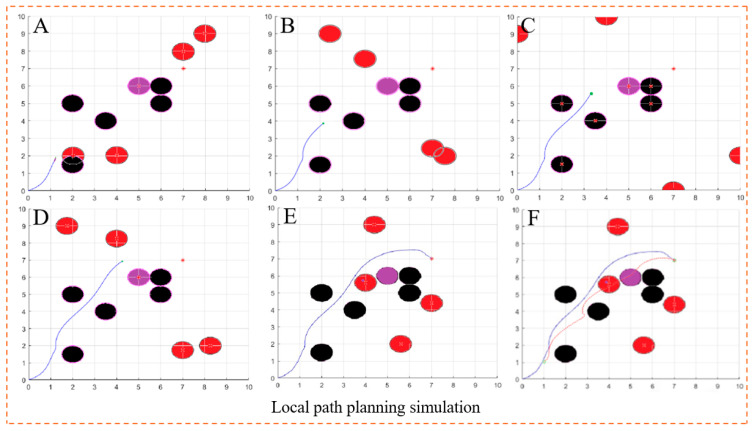
Simulation results of the improved dynamic window algorithm: (**A**,**B**) pre-operation diagram; (**C**,**D**) run-time diagram; (**E**,**F**) operation result diagram (the red part is the route planned by the artificial potential field algorithm).

Figure 8 shows the processing of different obstacles under various conditions when the dynamic window algorithm is run. The curve in a different color in Figure 8F is the global path planning curve obtained by the improved artificial potential field algorithm in the simulation experiment. When facing dynamic obstacles within the field of vision, the improved dynamic window algorithm selects the processing mode based on the speed of the dynamic obstacles, increasing the obstacle avoidance weight according to the sector distance, so that the AGV can travel safely. In the face of random static obstacles and upcoming dynamic obstacles, the AGV maintains a safe distance by reducing its speed by reducing its moving speed and finally successfully avoids all obstacles to reach the target point, which verifies the feasibility of the proposed algorithm.

## 9. Conclusions

To address the challenge of integrating both dynamic and static obstacles in a medical environment, this paper proposes an obstacle avoidance method for medical transport AGVs, inspired by the bionic characteristics of antelope migration. The approach combines an enhanced artificial potential field algorithm with the dynamic window algorithm. The concept of grafting is introduced to enhance the algorithm, analyzing the limitations and drawbacks of traditional obstacle avoidance methods. New obstacle avoidance strategies and an obstacle determination distance are incorporated into the original algorithm, followed by simulation experiments conducted in various environments using a numerical simulation platform. The experimental results demonstrate that the traditional artificial potential field algorithm fails to avoid obstacles and cannot reach the target point when faced with complex obstacles. The improved algorithm handles such scenarios more effectively. In the simulation example, the path planning success rate increased by 34%, the running time decreased by 33%, and the average path length was reduced by 1%. The introduction of the dynamic window algorithm for determining the sector distance expands the detection range, simultaneously avoiding both dynamic and static obstacles, and guiding the AGV toward safer path planning. The method proposed in this paper provides a theoretical foundation for improving the speed and safety of path planning for AGV-transported patients in a medical environment.

## Figures and Tables

**Table 1:** Main parameter settings of the improved artificial potential field algorithm.

Main Parameter	Magnitude
Gravitational gain coefficient (N/m)	28
Repulsion gain coefficient (N/m)	15
Obstacle evaluation radius (m)	0.4
AGV step length (m)	0.1

**Table 2:** Comparison of global path planning algorithm results.

Global Path Planning	Success Rate	Average Path Length (m)	Time Required to Reach the Target (s)
Traditional APF	66%	10.284	3.59 s
Improved APF	100%	10.191	2.40 s

**Table 3:** Main parameter settings of the improved dynamic window algorithm.

Main Parameter	Magnitude
Maximum speed (m/s)	1
Maximum rotation speed (rad/s)	2
Acceleration (m/s^2^)	3
Rotational acceleration (m/s^2^)	4
Speed resolution (rad/s)	0.1
Rotational speed resolution (rad/s)	0.1
Obstacle radius for conflict determination (m)	0.5

## Data Availability

Data used for this article may reasonably be obtained from the corresponding authors during the study period.
